# Sacro-coxxygial hygiene, a key factor in the outcome of pilonidal sinus surgical treatment?

**DOI:** 10.1186/s12893-021-01204-4

**Published:** 2021-04-17

**Authors:** Arnaud Dupuis, Niki Christou, Dorota Teterycz, Alexandre Balaphas, Joan Robert-Yap, Guillaume Zufferey, Karel Skala, Mariam Alketbi, Emilie Liot, Nicolas C. Buchs, Bruno Roche, Frederic Ris

**Affiliations:** 1 Department of Visceral Surgery, Geneva University Hospitals and Medical School, Rue Gabrielle-Perret-Gentil 4, 121, Geneva 14, Switzerland; 2 Service de Chirurgie Digestive, Générale et Endocrinienne, CHU de Limoges, Hôpital Dupuytren, 87042 Limoges Cedex, France; 3 Service de Chirurgie, Etablissements Hospitaliers du Nord Vaudois, Hôpital de la Vallée, Yverdon-les-bains, Switzerland; 4 Service de Chirurgie, Groupement Hospitalier de l’Ouest Lémanique, Nyon, Switzerland

**Keywords:** Pilonidal sinus, Limited excision, Hygiene, Recurrences, Sinusectomy surgical management of pilonidal sinus

## Abstract

**Background:**

Surgical wound infection contributes to prolonged recovery time after pilonidal sinus excision. As a standard procedure after surgery, we recommend our patients to perform water irrigations in the intergluteal cleft 4 to 6 times a day during the post-operative period. Our hypothesis is that this should reduce healing time and complication rates. The aim of this study was to measure the importance of sacro coccygeal hygiene in the management of pilonidal sinus disease.

**Methods:**

We retrospectively collected data after surgical management of pilonidal sinus (sinusectomy procedures) in our division over a 10-year period. Patients were divided into three groups according to their local hygiene during postoperative follow-up and scored one (G1: good hygiene) to three (G3: poor hygiene). Primary outcome was complication rates. Secondary endpoints were, healing time, follow-up, time off work, and recurrence rate.

**Results:**

In G1 (N = 112), complication rate was 3.6%. In G2 (N = 109), it was 5.5%, whereas in G3 (N = 71), it reached 7.03%. However, there were no statistically significant differences between hygiene groups regarding complication rates in both univariate and multivariable analysis. Regarding secondary outcomes, there were significant differences between hygiene groups concerning median follow-up (p = 0.0001) and median time off work (p = 0.0127).

**Conclusion:**

Good hygiene of wound is essential for optimal, rapid healing without complications. The importance of this report is to show that thanks to our hygiene follow-up strategy with frequent perineal irrigations and regular follow-up checks, patients with at a first glance “unclean local conditions”, reached similar complications, median healing time and recurrences rates to patients with medium and good wound hygiene level.

## Introduction

It is thought that the presence of hair in the gluteal cleft is responsible for pilonidal sinus disease [1]. This pathology was first described by Mayo in 1833 [2] and is probably an acquired disease [3, 4]. It is linked to the distention of a follicle with keratin which becomes inflamed and then obstructed [5]. It leads to infection with chronic non-healing sinus. Thus, presentation of pilonidal sinus disease encompasses various forms from chronic cysts with different sus-cutaneous tracts to acute cysts/sinus with abscess.

Pilonidal sinus disease usually occurs in young people between 15 and 30 years of age [5, 6] and concerns approximately 0.7% of the population [7]. Different predisposing factors have been highlighted in the literature. Some are “non-modifiable” such as age, ethnicity, gender, familial history of pilonidal disease, anatomy of the buttocks (thick skin, deep gluteal cleft…) whereas others can be modified: high density of hairs in the gluteal region, overweight, lack of hygiene, prolonged sitting, recurrent chafing [5].

Due to a large variety of treatments and lack of evidence, there is currently no consensus regarding pilonidal sinus disease management [8]. Published studies for the most part, have been heterogeneous regarding the techniques they used, underpowered, performed with a retrospective design or have short follow-ups [9].

**Fig. 1:**
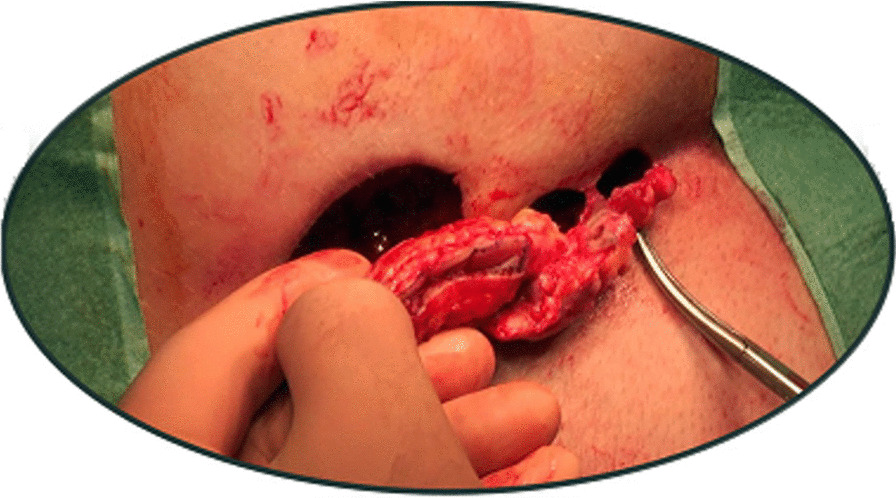
Pilonidal sinus excision—technique

First described by Lord-Millar [10], a limited excision of the cysts and sinus without skin closure was modified in our department by Prof Marc-Claude Marti. Skin bridges were left between each excision wound thus leaving a limited excision of the pilonidal sinus without skin closure (Fig. 1).

Bad personal hygiene has been described as a key factor in the development and recurrence of pilonidal sinus after surgery [11, 12].

The aim of the study was to assess the impact of post-operative sacro coccygeal hygiene on complication rates after pilonidal surgery.

## Methods

During a 10-year period, (01/07/2005 to 30/06/2015), we retrospectively included data from patients who had a limited excision of pilonidal sinus without skin closure in the Department of Visceral Surgery at the University Hospital of Geneva in Switzerland.

Patients below 16 years old, or pregnant, or with cognitive disorders and/or unable to follow the medical recommendations were excluded.

After surgery, good hygiene of the wound appeared to be a key factor to avoid complications and to promote rapid healing [12]. In our department, patients ‘wounds are closely monitored. During follow-up, wound characteristics are described in a standard form, using FileMaker software (FileMaker, Inc, Santa Clara, USA). Wound description is systematized with key words.

We looked for evidence of fibrin, inflammation, particles of soil, lint or hair and odour. The presence of each one was given a point and the total score then resulted in a grade of hygiene. Patients were classified and divided into three groups (which constitutes our “local grading system”) according to the surgeon’s assessment of local hygiene: G1 (good hygiene), G2 (moderate hygiene) and G3 (poor hygiene) (Table 1). It is worth to notice that the measurement of healing was also reported at each evaluation. It was determined by the percentage of epithelization of the wound, i.e. its percentage of closure. Since epithelialization is synonymous with fragility, quality of the epithelisation was determined by secondary absence of granulation tissue.

**Table 1:** Grades of hygiene. In italics, keywords used in the filemaker™ form

Grade	Fibrin	Inflammation/Pus	Particles/Stool	Smell	Score
G1	0	0	0	0	0
G2	1	1	1	0	1–3
G3	1	1	1	1	3–4

^1^If the wound was *non inflammatory*, rather *budding*, (Fig. 2)

^2^If it was *fibrinous*

^3^If the wound was *purulent* or with *stools* in it

**Fig. 2:**
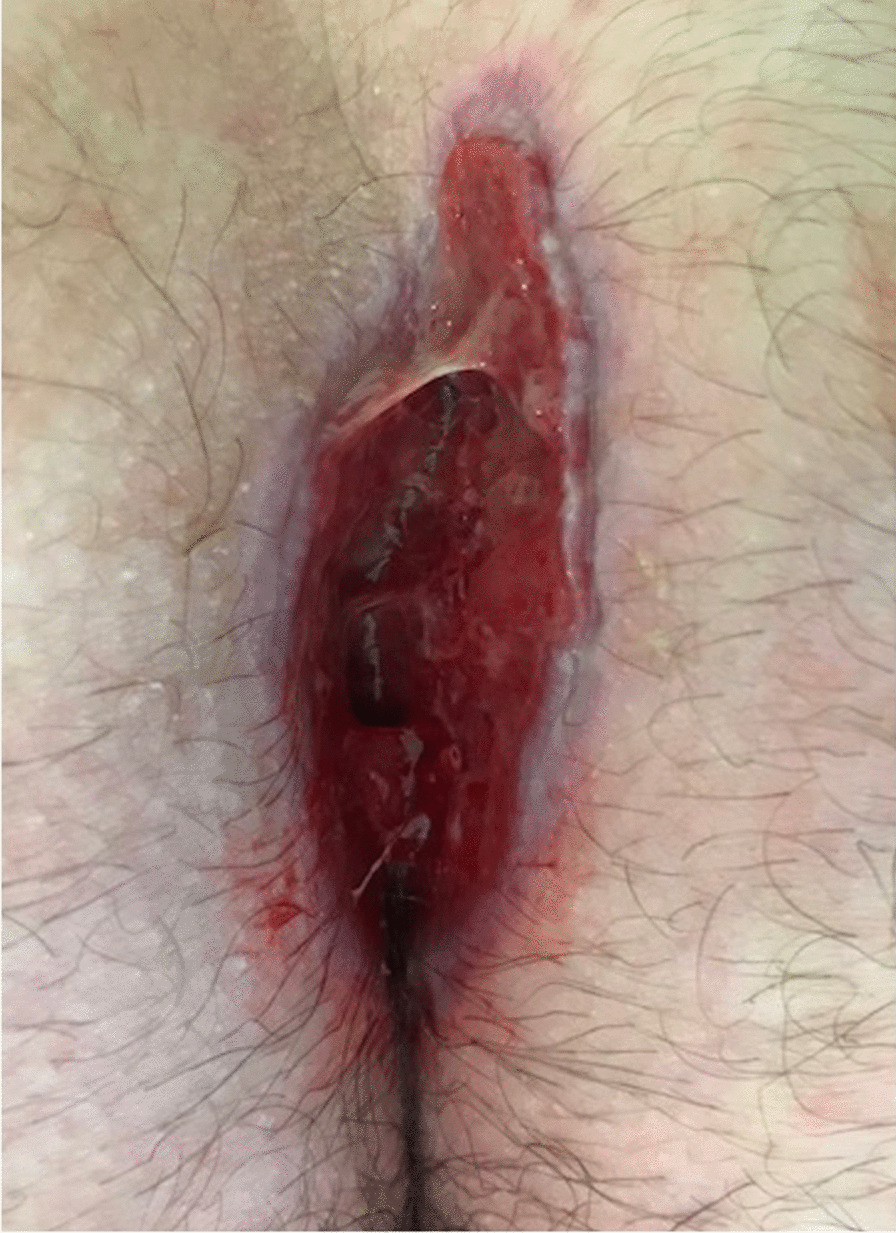
Pilonidal sinus, hygiene grade 1

This information is recorded at postoperative days 2 and 7. Patients are followed up to complete recovery (Fig. 2) and are advised according to their level of hygiene at each evaluation. In particular, we recommend sacro coccygeal irrigations (using a shower spray and only tap water) from four to six times a day during the initial post-operative period (Supplemental data, S1). In addition, patients were warned to avoid any tobacco or other poisoning (cannabis, etc.), to monitor their diabetes in order to balance it as well as possible, and follow the recommendations given by the medical staff concerning the wound at each visit. Moreover, when prominent sacral hairs were present, patients were shaved.

The primary endpoint was complication rates defined as the presence of abscess or bleeding. Secondary endpoints were recurrence rate, healing time, median follow-up, and median time off work.

### Statistical analysis

Power and sample size calculation was based on the logistic regression model, considering 10 patients for each term, a minimum recruitment of 60 patients was necessary which was far less than the total number of cases performed in our institution. Each variable was graphically evaluated to assess its distribution. Descriptive statistics were reported first. Chi 2, Fisher exact or Kruskal–Wallis two-sided tests were used to compare hygiene groups for primary and some secondary outcomes. Logistic regression was run in order to measure association between hygiene grades and outcomes and to eliminate potential confounding factors. These variables were directly included in the model. A p value < 0.005 was considered as statistically significant. Stata (StataCorp, College station, TX, USA). was used for all analyses.

## Results

Between July 2005 and June 2015, 377 patients were operated for pilonidal sinus disease; 292 of these were performed using the modified Lord-Millar technique (Table 1). The median age was 27 years old (15–59). There were 249 male and 43 female patients. The median number of primary orifices was 2 (1–16). Except for one, all patient had outpatient surgery. The procedure was performed under local anaesthesia in 94.5% of patients. Regarding wound hygiene, 112 patients were classified as G1, 109 as G2 and 71 as G3. Between different hygiene groups there were no statistically significant differences in terms of patient’s demographics (sex, median age). Interestingly, the median number of orifices was higher for G2 [2 (1–15)] and G3 [3 (1–7)] groups (p = 0.0001) (Table 2).

**Table 2:** Patients characteristics at the time of surgery according to hygiene grade

	G1 (N = 112)	G2 (N = 109)	G3 (N = 71)	p
Sex (H:F) (OR)	(91:21)	(92: 17)	(66:5)	0.089
Median age (range)	29 (16–59)	27 (15–57)	24 (16–58)	0.0779
Median number of primary orifices	2 (1–8)	2 (1–16)	3 (1–8)	0.0001
Anesthesia type: AL/AG	111/1	102/7	63/8	0.009

Complications rates did not significantly differ between hygiene groups (Table 3), in univariate (Table 4) and multivariable analysis (Table 5). Regarding secondary outcomes, such as median healing time and recurrence rates, there were no differences between the different groups of hygiene. But it is important to underline that the highest values (28 months for healing time and 9.2% for recurrences rates) were reached for patients with satisfactory hygiene grades (namely G1 and G2) (Table 3).

**Table 3:** Primary and secondary outcomes according to hygiene group

	G1 (N = 112)	G2 (N = 109)	G3 (N = 71)	p
Median healing time (range)	28 (5–123)	26 (5–95)	25 (8–98)	0.1685
Complications (%)	4/112 (3.57)	6/109(5.5)	5/71(7.04)	528
Median follow-up in months (range)	47.5 (1–124)	24 (1–76)	15 (3–82)	0.0001
Median Time off work (range)	12 (0–92)	11 (0–109)	14 (0–38)	0.0127
Recurrences rate (%)	4.46	9.17	8.45	0.357

**Table 4:** Univariate analysis of complication following pilonidal sinus disease surgery

Variables	Complications		
	Odds ratio	OR [95% CI]	p
Hygiene Grade 1	1		1
Hygiene Grade 2	1.57	[0.43–5.73]	0.493
Hygiene Grade 3	2.04	[0.53–7.89]	0.299

**Table 5:** Multivariate analysis of complication following pilonidal sinus disease surgery

Variables	Complications		
	Odds ratio	OR [95% CI]	p
Hygiene Grade 1	1		1
Hygiene Grade 2	1.47	[0.40–5.42]	0.558
Hygiene Grade 3	1.81	[0.46–7.11]	0.397
Age	0.99	[0.93–1.05]	0.759
Sex	0.44	[0.05–3.49]	0.438
Recurrences	1.83	[0.37–8.98]	0.454

The median follow-up was longer for grade of hygiene G1 [47.5 months (1–124)] (p = 0.0001) compared to other groups. There were also significant differences regarding median time off work (p = 0.0127) according to the hygiene grades (maximum of 14 days for G3). In univariate (Table 6) and multivariable analysis (Table 7), recurrence rate was the highest for G2 but no statistically significant differences were found between hygiene grades. Multivariate analysis was a logistic regression to measure association between hygiene grades and outcomes in order to avoid potential confounding factors.

**Table 6:** Univariate analysis of recurrence of pilonidal sinus disease after surgery

Variables	Recurrences		
	Odds ratio	OR [95% CI]	p
Hygiene Grade 1	1		1
Hygiene Grade 2	2.16	[0.71–6.54]	0.173
Hygiene Grade 3	1.97	[0.58–6.73]	0.277

**Table 7:** Multivariable analysis of recurrence of pilonidal sinus disease after surgery

Variables	Recurrences		
	Odds ratio	OR [95% CI]	p
Hygiene Grade 1	1		1
Hygiene Grade 2	1.92	[0.62–5.91]	0.255
Hygiene Grade 3	1.60	[0.46–5.58]	0.463
Age	0.93	[0.87–1.00]	0.039
Sex	0.63	[0.14–2.89]	0.554
Complications	0.32	[0.53–3.92]	0.478

## Discussion

In the literature, only one study by Mutus et al*.* was found to focus on wound infection after pilonidal sinus disease surgical treatment [13]. However, the authors studied a cohort of adolescents with a median age of 16 years old with a shorter follow-up (7 days–49 months) compared to our study (1–124 months). Their study showed that 36 patients out of 268 (13.4%) had wound complications, including infection and dehiscence, during the first month after surgery. Mutus et al*.* have a complication rate significantly higher than ours (whatever group of hygiene) which encompasses all complication types (bleeding, infection, dehiscence). We explain this difference by our strategy of close wound hygiene monitoring with small interventions at follow-up such as perineal/wound irrigation and/or perineal showers, fibrin removal, application of silver nitrate etc. These small interventions can influence cost management but will be always less expensive than the cost management of complications with in addition potential stress of another surgery.

Interestingly, significant differences of follow-up between hygiene groups were found with group G1 having the longest follow-up. However, the range obtained for G1 was the most important reflecting the possibility that patients in the initially G1 group, for lack of monitoring instructions (because G1 group significates to be at the least-risk hygiene group and therefore initially receiving fewer monitoring replicates and wound care instructions) were able to switch to a poorer hygiene group, resulting in an increase in the duration of the follow-up.

So far we could not find in the current literature mention of a hygiene score. As consequence, it has led our surgical team to direct the wound monitoring message to the clinic: poor hygiene groups were then encouraged to perform more showers and specific hygiene instructions were administered to them. Thus, recurrence rates between the different hygiene groups were similar. Furthermore, this highlights that the recognition of wounds’ dirt degree after surgery is predictive of the time of recovery (with significant difference of median time off work between hygiene groups) but did not alter the surgical technique.

In 2010, Mc Callum et al*.* [14], performed a Cochrane review to compare open versus closed surgical treatments of pilonidal sinus disease. No significant difference was found in the rate of infection between the two types of procedures (risk ratio 1.31, 95% CI 0.93–1.85). These results are difficult to compare with ours because they take into account several types of open surgical treatments, including the technique we are using.

Milone et al*.* [9] performed a review of the literature gathering 15 studies with different surgical approaches to treat pilonidal sinus disease. The overall incidence of recurrence of pilonidal sinus disease after surgery was 13.8% with a mean follow-up from 58.36 to 240 months. In this review, only 2 studies [15, 16] were focusing on sinusectomy such as in our study. These studies were published in 1995 (Matter et al.) [15] and 2008 (Gips et al*.*) [16]. Matter et al*.* included 50 patients with a mean age of 25 years old, with a mean follow-up of 72 months and described 18 recurrences (36%). The study of Gips et al. [16] included 1165 patients and reported 189 recurrences (16.2%) for a mean follow-up of 82.8 months. These recurrence rates are higher than the incidence of 7.2% we reported in this study with a median follow-up of 24 months.

The lowest and highest recurrence rates by grade of wound hygiene in the present study were found in respectively G1 (4.5%) and G2 (9.2%). These results have to be compared to a 2010 Cochrane review including studies with different surgical approaches were the recurrence rate for open healing approach (without cutaneous bridges) was 5.3% and global recurrence rate was 7% [8].

This study has some drawbacks. Firstly, the design was retrospective and based on records of wound aspects described by key words for the purpose of documenting the patient’s visit on his file and not to collect data for a study. We did not have the possibility to retrieve the name of surgeons who evaluated each patient. However, due to the limited number of surgeons involved in patient follow-up, the variability between observers would be low. Secondly, some important and potentially confounding variables were missing in patients’ files, such as diabetes, smoking, BMI etc. Finally, wound hygiene advises that were probably tailored to the aspect of the wound could have influenced wound classification. Thus, this report should be completed with a prospective study with direct classification of wound aspect according to the grade we used and assessor training with wound pictures. Moreover, all variables of interest should be collected.

Many studies have focused on the priority of hair removal after surgery. A recent literature review published in 2018 by Pronk et al*.* [17] has underlined lower recurrence rates of pilonidal disease after laser hair removal compared to other methods of hair removal. However, the quality of methodology was limited. It would be interesting to incorporate in a future score, hair removal characteristics (extent of hair removal, technique etc.).

Wound hygiene is predictive of infection [18]. So far we could not find in the current literature mention of wound hygiene classification after pilonidal sinus disease surgery. We herein demonstrated that with a strategy of close wound monitoring and feedback interventions on wound hygiene we reach lower complication rates compared to the current literature and similar outcomes of complications and recurrence for dirty and clean wounds.

## Conclusion

There is little evidence in literature that one surgical technique is better than another for pilonidal sinus disease. The originality of our centre in the postoperative care of patients is the strategy of close wound monitoring. This interaction has led to a positive management of wounds with sacro coccygeal irrigations. As a result, there were no statistical differences in terms of complications between hygiene groups. This study highlighted the level of wound cleanliness after surgery is a key point to avoid complications. A future prospective study comparing regular washing plus hair removal versus washing alone needs to be implemented.

## Data Availability

Database is at the disposal of the journal if required: you can contact Dr CHRISTOU Niki, corresponding author at christou.niki19@gmail.com.

## References

[1] Hull TL, Wu J (2002). Pilonidal disease. Surg Clin.

[2] Mayo OH (1833). Observations on injuries and diseases of the rectum.

[3] Søndenaa K, Andersen E, Nesvik I, Søreide JA (1995). Patient characteristics and symptoms in chronic pilonidal sinus disease. Int J Colorectal Dis.

[4] Karydakis GE (1992). Easy and successful treatment of pilonidal sinus after explanation of its causative process. Aust N Z J Surg.

[5] Doll D, Friederichs J, Dettmann H, Boulesteix A-L, Duesel W, Petersen S (2008). Time and rate of sinus formation in pilonidal sinus disease. Int J Colorectal Dis.

[6] Shabbir J, Chaudhary BN, Britton DC (2011). Management of sacrococcygeal pilonidal sinus disease: a snapshot of current practice. Int J Colorectal Dis.

[7] AL‐Khamis A, McCallum I, King PM, Bruce J. Healing by primary versus secondary intention after surgical treatment for pilonidal sinus. Cochrane Database Syst Rev [Internet]. 2010 [cited 2018 Dec 5];(1). https://www.cochranelibrary.com/cdsr/doi/10.1002/14651858.CD006213.pub3/abstract. 10.1002/14651858.CD006213.pub3 PMC7055199 20091589

[8] Milone M, Velotti N, Manigrasso M, Anoldo P, Milone F, De Palma GD (2018). Long-term follow-up for pilonidal sinus surgery: a review of literature with metanalysis. The Surgeon.

[9] Lord PH, Millar DM (1965). Pilonidal sinus: a simple treatment. BJS.

[10] Bascom J (1980). Pilonidal disease: origin from follicles of hairs and results of follicle removal as treatment. Surgery.

[11] Sevinç B. Treatment of pilonidal disease. 2017;1–4.

[12] Mutus HM, Aksu B, Uzun E, Gulcin N, Gercel G, Ozatman E (2018). Long-term analysis of surgical treatment outcomes in chronic pilonidal sinus disease. J Pediatr Surg.

[13] McCallum I, King PM, Bruce J, AL‐Khamis A. Healing by primary versus secondary intention after surgical treatment for pilonidal sinus. Cochrane Database Syst Rev [Internet]. 2007 [cited 2018 Dec 5];(4). https://www.cochranelibrary.com/cdsr/doi/10.1002/14651858.CD006213.pub2/abstract. 10.1002/14651858.CD006213.pub2 17943897

[14] Matter I, Kunin J, Schein M, Eldar S (1995). Total excision versus non-resectional methods in the treatment of acute and chronic pilonidal disease. BJS.

[15] Gips M, Melki Y, Salem L, Weil R, Sulkes J (2008). Minimal surgery for pilonidal disease using trephines: description of a new technique and long-term outcomes in 1,358 patients. Dis Colon Rectum.

[16] Pronk AA, Eppink L, Smakman N, Furnee EJB (2018). The effect of hair removal after surgery for sacrococcygeal pilonidal sinus disease: a systematic review of the literature. Tech Coloproctology.

[17] Allegranzi B, Bischoff P, de Jonge S, Kubilay NZ, Zayed B, Gomes SM (2016). New WHO recommendations on preoperative measures for surgical site infection prevention: an evidence-based global perspective. Lancet Infect Dis.

[18] Andersen BM. Prevention of postoperative wound infections. In: Andersen BM, (ed). Prevention and control of infections in hospitals: practice and theory [Internet]. Cham: Springer International Publishing; 2019 [cited 2019 Apr 12]. p. 377–437. 10.1007/978-3-319-99921-0_33.

